# Dorso‐Ventral and Night‐Day Regulation of Extracellular K^+^ Dynamics in Mouse Hippocampal Astrocytes

**DOI:** 10.1002/glia.70201

**Published:** 2026-07-09

**Authors:** Nariman Kiani, Kabeer Abubakar, Tin Luka Petanjek, Monique Esclapez, Anton Ivanov, Christophe Bernard

**Affiliations:** ^1^ Aix Marseille Univ, INSERM, INS, Inst Neurosci Syst Marseille France; ^2^ Department of Human Anatomy, Faculty of Basic Medical Sciences Federal University of Lafia Lafia Nigeria; ^3^ Department of Physiology and Department of Neuroscience, Croatian Institute for Brain Research, School of Medicine University of Zagreb Zagreb Croatia; ^4^ Aix Marseille Univ, Inserm INMED, Institut de Neurobiologie de la Méditerranée Marseille France

**Keywords:** astrocytes, circadian rhythm, hippocampal regions, K+ homeostasis, Kir4.1 channels

## Abstract

Astrocytes regulate extracellular potassium (K^+^) through multiple mechanisms operating across distinct spatiotemporal scales, yet whether this regulation exhibits regional and circadian specificity remains unclear. Using real‐time K^+^ measurements combined with electrophysiology and immunohistochemistry in astrocytes, we characterize K^+^ buffering by astrocytes across the dorsoventral hippocampal axis at three circadian times. We report that hippocampal CA1 *stratum radiatum* astrocytes possess different functional K^+^ buffering capacities depending on both anatomical location and time of day. At the early light phase (ZT3), the ventral hippocampus (VH) exhibits faster K^+^ accumulation and greater peak amplitudes than the dorsal hippocampus (DH) in response to similar neuronal network activity. This regional divergence is driven by reduced Kir4.1 channel function in VH astrocytes, which persists across all measured time points, and which is partially compensated by enhanced Na^+^/K^+^‐ATPase activity specifically at ZT3. Gap junction coupling between astrocytes shows regional and time‐dependent variation, with elevated coupling in VH at ZT3 that subsequently normalizes. Kir4.1 protein expression exhibits circadian dynamics. In contrast to the steady, high levels of Kir4.1 observed in the DH, the VH shows a gradual increase in expression across the light–dark cycle. These findings establish astrocytic K^+^ buffering as a multiscale phenomenon integrating regional heterogeneity with circadian regulation, with implications for understanding regional and temporal differences in network excitability, particularly in epilepsy.

## Graphical abstract

Hippocampal astrocytic K^+^ buffering varies by region and time. Ventral astrocytes show faster K^+^ accumulation due to reduced Kir4.1 function. Such regional divergence is modulated by circadian shifts in Kir4.1 and gap junction coupling.

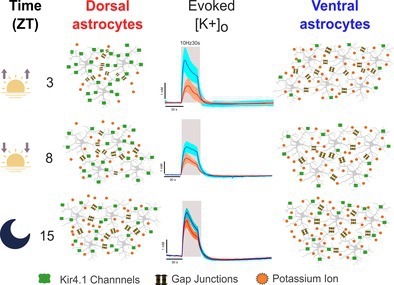

## 1 Introduction

Under physiological conditions, extracellular K^+^ concentration ([K^+^]_o_) fluctuates between 2 and 5 mM, while increases above 10 mM are linked to pathological activities such as seizures and spreading depression (de Curtis et al. 2018; Enger et al. 2015; Erecińska and Silver 1994). Maintaining [K^+^]_o_ within narrow physiological limits is therefore functionally important.

Astrocytes play an important role in regulating [K^+^]_o_ by taking up excess extracellular K^+^, redistributing it through gap‐junction–connected networks, and extruding it where [K^+^]_o_ is lower (Wallraff et al. 2006; Walz 2000). This process relies on inward‐rectifying Kir4.1 channels (Seifert et al. 2009), and the Na^+^/K^+^‐ATPase pump. Impairment of either component can increase neuronal and network activity and excitability (Djukic et al. 2007; Romanos et al. 2020; Wallraff et al. 2006).

Neuronal activity and excitability are not uniform across the brain. Even within a single structure, distinct subregions exhibit marked differences in intrinsic excitability, synaptic properties, and responses to pharmacological or pathological challenges. For example, along the dorsoventral axis of the hippocampus, the ventral region (VH) displays higher intrinsic excitability, greater seizure susceptibility, distinct patterns of synaptic plasticity, and different metabolic properties compared to the dorsal region (DH) (Brancati et al. 2021; Dougherty et al. 2012; Papatheodoropoulos et al. 2005). Similarly, neuronal activity is not constant across time: neuronal excitability, synaptic transmission, and seizure threshold change during the night and day cycle (Chang, Leite, et al. 2018; Debski et al. 2020; Quigg et al. 2000), reflecting temporal regulation of molecular, metabolic, and physiological processes (Brancati et al. 2021; Debski et al. 2020; McCauley et al. 2020).

This raises a fundamental question: do astrocytes exhibit similar spatial and temporal heterogeneity in their K^+^ buffering capacity? Do they “follow the pace” of neuronal activity, adapting their homeostatic functions to match regional differences in excitability and circadian variations in network state? If astrocytic K^+^ buffering were spatially and temporally invariant, regions of high neuronal excitability (such as the VH) and periods of elevated seizure susceptibility (such as specific circadian phases) would be vulnerable to inadequate K^+^ homeostasis. Conversely, if astrocytes dynamically adjust their buffering capacity in space and time, this could represent a sophisticated form of adaptive homeostasis matched to local circuit demands.

The hippocampus provides an ideal system to address this question. Beyond the well‐documented differences in neuronal properties along the dorsoventral axis and across circadian time, the hippocampus also exhibits regional and temporal heterogeneity in astrocytic molecular markers, morphology, and calcium signaling (Dong et al. 2009; Jinno 2011; Ryu et al. 2024; Thompson et al. 2008). However, whether these molecular and morphological differences translate into functional differences in K^+^ buffering—and how they relate to the known regional and circadian variation in neuronal excitability—remains unclear.

Here, we directly test whether astrocytic K^+^ buffering capacity varies across the dorsoventral hippocampal axis and across circadian time, using integrated functional, electrophysiological, and molecular approaches. We use an extracellular K^+^ sensor to measure K^+^ dynamics during standardized synaptic stimulation (Schaffer collateral activation), monitoring multiple parameters of the K^+^ transient (accumulation rate, peak amplitude, clearance kinetics, and Na^+^/K^+^‐ATPase‐dependent undershoot) in dorsal and ventral hippocampal slices at three Zeitgeber times: ZT3 (early light, baseline period), ZT8 (late light, period of heightened seizure susceptibility) (Chang, Kudlacek, et al. 2018; Debski et al. 2020; Quigg et al. 2000), and ZT15 (early dark, active phase). We then employ whole‐cell patch‐clamp electrophysiology to assess the cellular mechanisms underlying these functional differences, examining gap‐junction coupling, Kir4.1 channel function, and astrocyte resting membrane potential. Finally, we use immunohistochemistry to quantify regional and circadian variation in Kir4.1 protein expression. Our findings demonstrate that astrocytic K^+^ buffering capacity depends upon both anatomical location and circadian phase, revealing a form of adaptive heterogeneity that parallels—but does not simply mirror—the spatial and temporal organization of neuronal excitability.

## 2 Methods

### 2.1 Animals and Ethical Approval

All animal procedures were conducted in accordance with the European Directive 2010/63/EU and French regulations on animal experimentation. The study protocol was approved by the relevant institutional and national ethics committees (APAFIS authorization #44598‐2023081812555435v4). A total of 59 male FVB mice (weight: 28–45 g; age: 2 months or older) were used. Of these, 12 mice were dedicated to immunohistochemistry, while the remaining animals were used for both patch‐clamp experiments and K^+^ dynamic recordings in parallel. The single‐sex design increased statistical power and ensured an adequate yield of viable samples given the technically demanding nature of the experiments. The exclusion of female mice is a limitation of the study, and future work will be required to assess the generalizability of these findings across sexes.

### 2.2 Brain Slice Preparation

Mice anesthetized with sevoflurane, were decapitated, and the brain was swiftly removed from the skull and submerged in ice‐cold artificial cerebrospinal fluid (ACSF) (in mM): NaCl 126, KCl 3.50, NaH_2_PO_4_ 1.25, NaHCO_3_ 25, CaCl_2_ 2, MgSO_4_1.3, and glucose 10, with a pH value maintained between 7.3 and 7.4. The ACSF was aerated with a 95% O_2_/5% CO_2_ gas mixture. One hemisphere, left or right in alternation, was used to prepare dorsal slices via coronal sections, whereas the other hemisphere was used to prepare ventral slices as described previously (Dougherty et al. 2012). The brain was immersed in an ice‐cold cutting solution (in mM): K‐gluconate 140, HEPES 10, Na‐gluconate 15, EGTA 0.2, and NaCl 4, with the pH adjusted to 7.2 with KOH. Using a tissue slicer (Leica VT 1200s, Leica Microsystem, Germany), 300 μm‐thick for patch‐clamp recording and 350 μm‐ thick slices for field recording were produced. Subsequently, to preserve the cells near the surface from resulting damage caused by slicing, the 300 μm‐thick slices were incubated for 15–20 min at room temperature (RT) in a choline chloride solution (in mM): Choline Chloride 110, KCl 2.5, NaH_2_PO_4_ 1.25, MgCl_2_ 10, CaCl_2_ 0.5, NaHCO_3_ 25, Glucose 10, Na‐Pyruvate 5, constantly bubbled with a 95% O_2_/5% CO_2_ gas mixture. To label the astrocytes, the slices were incubated for 20 min in a ACSF solution containing 1 μM of sulforhodamine 101 (SR101, Merk), a fluorescent dye specifically absorbed by astrocytes and not neurons (Nimmerjahn et al. 2004). The slices were allowed to recover in a dual‐side perfusion holding chamber with continuously circulating ACSF for at least one hour before experimentation.

### 2.3 Synaptic Stimulation and Field Potential Recordings

In parallel, 350 μm‐thick transferred to a dual perfusion recording chamber continuously perfused (~6 mL/min) with ACSF containing 5 mM of glucose, warmed to 30°C. Schaffer collateral (SC) was stimulated with a bipolar metal electrode connected to a DS2A‐isolated stimulator (Digitimer Ltd., UK). The electrode tip was placed in the *Stratum Radiatum* (*s.r*.) between the CA3 and CA2 regions. Extracellular recording electrodes were pulled (Harvard Apparatus Ltd., UK, O.D. = 1.2 and I.D. = 0.94 mm, Rp < 1 MΩ) using a horizontal puller (Sutter Instruments, USA). The extracellular local field potential (LFP) was recorded with a glass microelectrode filled with ACSF, placed in the *Stratum Oriens* (*s.o*.) toward the *Stratum Pyramidale* (*s.p*.) of the CA1 area. The signal was amplified 1000 times with an EXT‐02F amplifier (NPI Electronic, Germany) operating with a low‐pass 3 kHz filter (DC mode). The stimulation current intensity was adjusted to produce an LFP response of 60%–70% of the maximum amplitude, equivalent to 80–170 μA. Each slice was stimulated with trains of stimulation pulses of different frequencies ranging from 2.5 to 100 Hz. The LFP responses were recorded simultaneously with the K^+^‐dependent potential using Patch Master software. Each stimulation protocol was repeated twice. The LFP responses to single pulse stimulation were monitored in between train stimulations to check the slice stability. If the response decreased by more than 20%, the slice was discarded. In this study, we used the response to the 10Hz30s trains because this stimulation evoked a K^+^ signal with good SNR, allowing the measurement of required parameters (peak amplitude, 25%–75% rise time, decay time, and undershoot amplitude) of the individual and not the averaged response.

### 2.4 Fabrication of Potassium Sensitive Microelectrode

K^+^‐selective microelectrodes were prepared using the method described by Heinemann and Arens (1992). In brief, electrodes were pulled from double‐barrel theta glass (TG150‐4, Warner Instruments, Hamden, CT, USA). The reference barrel was filled with 154 mmol/L NaCl solution. The silanized ion‐sensitive barrel tip (5% trimethyl‐1‐chlorosilane in dichloromethane) was filled with a potassium ionophore I cocktail A (60,031 Fluka distributed by Sigma‐Aldrich, Lyon, France) and backfilled with 100 mmol/L KCl. Measurements of K^+^‐dependent potentials were performed using a high‐impedance differential DC amplifier equipped with negative capacitance feedback control, which permitted the compensation of the microelectrode capacitance required for the recording of the rapid changes of [K^+^]_o_. The electrodes were calibrated before each experiment. The calibration curve, concentration versus potential, was fitted with an exponential function. The obtained equation was used to convert [K^+^]_o_‐dependent potential recordings into concentration changes.

### 2.5 Electrophysiological Recording of the Astrocytes

For the electrophysiological recordings of astrocytes, 300 μm‐thick slices were immersed in a low‐volume (2 mL) recording chamber and continuously perfused with ACSF at 32°C–34°C and a perfusion rate of 5 mL/min. Sulforhodamine labeled astrocytes in the *stratum radiatum* (*s.r*.) of the CA1 region of the hippocampus were identified as a fluorescent cells with astrocytic morphology (excitation/emission wavelengths of 586/605 nm). Patch pipettes were fabricated using the same horizontal puller from borosilicate glass tubing (Harvard Apparatus Ltd., UK,1.5 mm outer diameter (O.D.), 0.86 mm inner diameter (I.D.), pipette resistance (Rp) = 5.78 ± 0.12 MΩ) and filled with an intrapipette solution (in mM): KCl 20, K‐gluconate 115, HEPES 10, EGTA 1.1, MgATP 4, Na‐phosphocreatine 10, Na_2_GTP 0.4, and 0.3%–0.4% biocytin (Sigma, Germany). Signals were transmitted to a Multiclamp 700A amplifier (Molecular Devices), digitized at a rate of 10 kHz using a DigiData 1550 interface (Molecular Devices) connected to a personal computer, and analyzed using ClampFit software (Molecular Devices). The resting membrane potential (RMP) of astrocytes was measured using the patch‐clamp technique, whole‐cell configuration, in current clamp mode.

### 2.6 Isolation of Kir4.1 Currents

To isolate Kir4.1 currents and to keep constant the extracellular K^+^, slices were incubated for 36 ± 2 min in the baseline 5 mM glucose‐containing ACSF supplemented with (in μM): Meclofenamic acid (MFA, Sigma‐Aldrich) 100 to block gap junctions, and Tetrodotoxin (TTX, Abcam) 0.5, 6‐Cyano‐7‐nitroquinoxaline‐2,3‐dione (CNQX, HelloBio) 10, and D‐(−)‐2‐Amino‐5‐phosphonopentanoic acid (D‐AP5, HelloBio) 40 to stop neuronal firing and minimize synaptic transmission. No effect of incubation time on Kir4.1 current was detected. Upon establishment of the whole‐cell patch clamp configuration, the patched astrocyte was held at −70 mV in voltage clamp mode, and after 5 min of dialysis, membrane currents were recorded at different holding potentials varying from −140 to 50 mV with 10 mV steps. Then, to block Kir4.1 channels, 100 μM of BaCl_2_ was added to the baseline ACSF, and the currents elicited by the same voltage steps were recorded after 5 min. The current–voltage relationship of Ba^2+^ sensitive conductance was determined by subtracting the currents recorded in Ba^2+^ from those recorded before its administration. Access resistance was determined by biexponentially fitting the capacitive current resulting from −70 to −60 mV voltage step. Cells were discarded if they met any of the following criteria: an initial access resistance above 35 MΩ, a resting membrane potential (RMP) less negative than −50 mV, or a final access resistance increase of more than 20%.

### 2.7 Coupling Estimation

Astrocytes are extensively coupled via gap junctions formed by Cx43 and Cx30, supporting intercellular redistribution of ions and metabolites, including potassium buffering. Pharmacological inhibition of these gap junctions is expected to uncouple astrocytes and thereby reduce their capacity for spatial potassium buffering. In principle, comparing K^+^‐dependent currents evoked by Schaffer collateral stimulation before and after gap junction blockade could provide an estimate of the coupling state of the recorded astrocyte.

However, available gap junction inhibitors lack specificity and profoundly affect neuronal and glial function. Carbenoxolone alters both AMPA and GABA_A_ receptor‐mediated synaptic transmission (Tovar et al. 2009). Meclofenamic acid is a potent activator of KCNQ2/Q3 (Kv7.2/7.3, M‐type) channels (Anderson et al. 2009). and, at the 50–100 μM concentrations commonly used to block gap junctions, hyperpolarizes CA1 neurons and suppresses synaptic responses (Peretz et al. 2005). Connexin mimetic peptides such as Gap26 and Gap27, while they are more specific at the channel level, also reduce basal excitatory synaptic transmission, likely by inhibiting gliotransmitter release through Cx43 hemichannels (Dospinescu et al. 2025). Because all of these agents can alter overall network activity, they inevitably affect not only K+ buffering but also activity‐dependent K+ release. To avoid these confounding effects, we adopted an alternative approach to assess astrocytic coupling, based on the spread of the gap junction–permeable intracellular tracer biocytin following controlled whole‐cell loading (Stephan et al. 2021).

In a separate set of experiments, astrocytes were patched in the absence of any blocker in whole cell configuration and kept at −70 mV holding potential for 20 mins. Slices were then fixed in 4% paraformaldehyde (AntigenFix, Diapath) overnight at 4°C. After three 30‐min washes in 0.12 M phosphate buffer (PB), slices were cryoprotected in 20% sucrose overnight, then deep‐frozen on dry ice and stored at −80°C until the day of experimentation.

To reveal biocytin‐containing astrocytes, slices were rinsed in PB twice, in 0.02 M potassium phosphate buffered saline (KPBS) three times for 30 min, and then incubated with streptavidin conjugated with fluorophore Alexa 488 (Abcam) diluted (1/200) in KPBS containing 0.3% Triton‐X100, overnight at RT. After washing with KPBS for 3 × 30 min, slices were mounted on Superfrost slides using Fluoromount‐G.

To visualize the stained astrocytes coupling, slices were imaged using a Zeiss LSM 700 inverted confocal microscope with a 20× (0.5× zoom) objective. Z‐stacks (15–35 optical slices, length: 2048 pixels, width: 2048; 1 μm interval) were acquired in the CA1 *stratum radiatum* using Zeiss ZEN software. Z‐stacks were merged using Fiji software (NIH), and pixel intensity was then normalized.

A blinded examiner then counted the number of stained astrocytes. Since only one astrocyte per slice was patched, the stained astrocytes were mandatory, those connected with the patched one directly or through other connected astrocytes. Therefore, we considered the number of stained astrocytes as an estimation of the astrocytic coupling.

### 2.8 Immunohistochemistry and Image Analysis

#### 2.8.1 Tissue Preparation, Immunohistochemistry, and Image Analysis

Wild‐type male FVB mice (P50–70) were anesthetized with an intraperitoneal injection of ketamine and xylazine, followed by a lethal dose of pentobarbital administered at designated Zeitgeber times (ZT3, ZT8, or ZT15). Mice were then transcardially perfused with cold 0.12 M PB to remove the blood and then with AntigenFix to fix the tissue. Brains were extracted, post‐fixed for up to 4 h in the same fixative, rinsed in PB, cryoprotected in 20% sucrose in PB overnight at 4°C, quickly frozen on dry ice and kept at −80°C. Hemisected blocks of brain containing dorsal and ventral hippocampus were sectioned at 40 μm using a cryostat. The Sections were collected sequentially in tubes containing an ethylene glycol‐based cryoprotective solution (Lu and Haber 1992; Watson et al. 1986) and stored at −20°C until histological processing.

#### 2.8.2 Double Labeling for GFAP and Kir4.1

Sections were processed for the simultaneous detection of GFAP to label astrocytes and the Kir4.1 channel. They were rinsed for 30 min in PB and for 2 × 30 min in KPBS. Sections were then incubated for 1 h at RT in 3% normal donkey serum (NDS) diluted in KPBS containing 0.3% Triton X‐100 and overnight at RT in a solution containing the polyclonal guinea pig anti‐GFAP (1:600, #AFP‐001‐GP, Alomone Labs) and polyclonal rabbit anti‐Kir4.1 (1:500, #APC‐035, Alomone Labs) diluted in KPBS containing 0.3% Triton X‐100 and 1% NDS. After 3, 30‐min rinses in KPBS, sections were incubated for 2 h in Alexa Fluor 488‐conjugated donkey anti‐guinea pig IgG (1:100, #706‐545‐148, Jackson ImmunoResearch) and Cy3‐conjugated donkey anti‐rabbit IgG (1:100, #711‐167‐003, Jackson ImmunoResearch), diluted in KPBS and 3% NDS. Sections were then rinsed with 3 × 30 rinses of KPBS and mounted on Superfrost slides using Fluoromount‐G containing the nuclear marker DAPI (Thermo Fisher).

Immunofluorescence was imaged on a Zeiss LSM 700 inverted confocal microscope using a 63× oil‐immersion objective. Z‐stacks (12–19 optical slices at 1 μm intervals) of the CA1 *stratum radiatum* were acquired using Zeiss ZEN software. One field of view per section was imaged. Z‐projections were performed in Fiji (NIH), and GFAP signal was quantified by measuring its integrated density. Thereafter the GFAP images were normalized (on maximum or minimum or mean intensity), thresholded, and then binarized. After removing outlier pixels, a mask was created to define the region of interest (ROI) for Kir4.1 analysis. Then, Kir4.1 signal within the GFAP‐defined ROI was quantified using the AND operation in the Image Calculator, and the Kir4.1/GFAP integrated density ratio was calculated (Bataveljic et al. 2024).

### 2.9 Statistical Analysis

All statistical analyses were performed in R (version 4.5.1). Our goal was to quantify the magnitude and uncertainty of regional and circadian effects on astrocytic and network measures while making transparent how inferences depend on reasonable analytic choices. To this end, we combined permutation‐based hypothesis testing with Bayesian multilevel modeling and a limited number of clearly labeled pairwise comparisons. For each outcome, we pre‐specified a primary inferential framework and used additional analyses as robustness checks and descriptive follow‐ups rather than as independent confirmatory tests (Yaddanapudi 2016).

#### 2.9.1 Primary Analyses: Permutation ANOVA

For outcomes defined at the slice or cell level and measured across hippocampal regions (dorsal vs. ventral), Zeitgeber time (ZT3, ZT8, ZT15), and their interaction, we first assessed global effects using a non‐parametric permutation ANOVA (Freedman–Lane procedure, 10,000 permutations) implemented in the permuco package (Kherad‐Pajouh and Renaud 2015). This approach was chosen to provide robust inference with minimal assumptions about normality, homoscedasticity, or sphericity while respecting the factorial structure of the design. We report permutation‐based *p* values together with ω^2^ as an effect‐size measure, representing the proportion of variance in the population attributable to each factor. The magnitude of regional differences was also evaluated using an online estimation statistics tool, which reported the mean difference and corresponding 95% confidence intervals (Ho et al. 2019).

Permutation ANOVA results are used to address global questions about whether region, time, or their interaction influence a given variable (e.g., K peak amplitude, decay time constant, undershoot amplitude, gap‐junction coupling, Kir4.1 expression). When a main effect or interaction was supported by the permutation test, we examined the corresponding pattern of estimated effects in the Bayesian models described below, focusing on the consistency of direction and the size and uncertainty of the effects.

#### 2.9.2 Primary Analyses for Hierarchical Data: Bayesian Multilevel Modeling

Because several outcomes involve hierarchical structure (multiple cells nested within animals and/or slices) and may deviate from Gaussian distributions, we complemented the permutation ANOVA with Bayesian multilevel models fitted using brms (version 2.22.0) (Bürkner 2017). For each variable, we used the fitdistrplus package to select an appropriate likelihood family based on information criteria, ensuring that the model captured the empirical distribution of the data (Delignette‐Muller and Dutang 2015). Models included fixed effects of region, Zeitgeber time, and their interaction, with random intercepts for animal (and slice when applicable) to account for within‐animal dependence.

Bayesian models were fitted with four MCMC chains (4000 iterations per chain, 2000 warm‐up), and convergence was confirmed by $\overset{`}{R}\leq 1.01$ and adequate effective sample sizes. We used weakly informative priors centered on no effect, chosen to regularize extreme estimates without imposing strong prior beliefs. For each parameter, we report the posterior median and 95% credible interval (CrI). In line with recent recommendations, we do not dichotomize effects based on arbitrary thresholds but instead interpret the magnitude and uncertainty of the posterior estimates in light of the permutation results and the broader pattern across variables.

For variables with substantial hierarchical structure (e.g., gap‐junction coupling counts, Kir4.1 currents, and some electrophysiological measures), the Bayesian multilevel models are considered the primary analyses because they explicitly model within‐animal clustering and non‐Gaussian distributions. In these cases, permutation ANOVA is treated as a robustness check that provides a complementary non‐parametric assessment of global effects.

#### 2.9.3 Pairwise Comparisons and Multiple Testing

Instead of relying on *p*‐vlaue thresholds, we evaluate the magnitude and certainty of regional and circadian differences using the posterior distributions. Parameter estimates are reported as posterior medians alongside 95% Highest Posterior Density (HPD) credible intervals. We consider an effect to be robust if its 95% HPD interval reliably excludes zero. To limit inflation of type I error across multiple time‐point–specific comparisons, we applied the Bonferroni method within each logical family of exploratory tests. (e.g., the set of dorsal–ventral contrasts across ZT for a given variable). Therefore, our main conclusions do not rely on isolated uncorrected pairwise differences, but on the pattern of effects supported by the primary global models and their associated effect‐size and uncertainty estimates.

#### 2.9.4 Interpretation of Borderline and Analysis‐Dependent Results

Following current recommendations on the use of *p* values, we do not treat crossing the conventional threshold of 0.05 (e.g., *p* = 0.0496) as a qualitative boundary between the presence and absence of an effect. Instead, borderline *p* values are interpreted alongside effect sizes, confidence intervals, and the corresponding Bayesian posterior estimates. When permutation ANOVA and Bayesian multilevel modeling yield discordant conclusions, we explicitly label the finding as analysis‐dependent and interpret it as tentative. Such cases are discussed as hypotheses that warrant further confirmation rather than as central results. Statistical significance is only denoted when Bonferroni correction and the credible intervals agree.

## 3 Results

### 3.1 Extracellular K^+^ Dynamics Exhibit Regional and Circadian Specificity

#### 3.1.1 Synaptic Strength Is Comparable Across Space and Time

**FIGURE 1:**
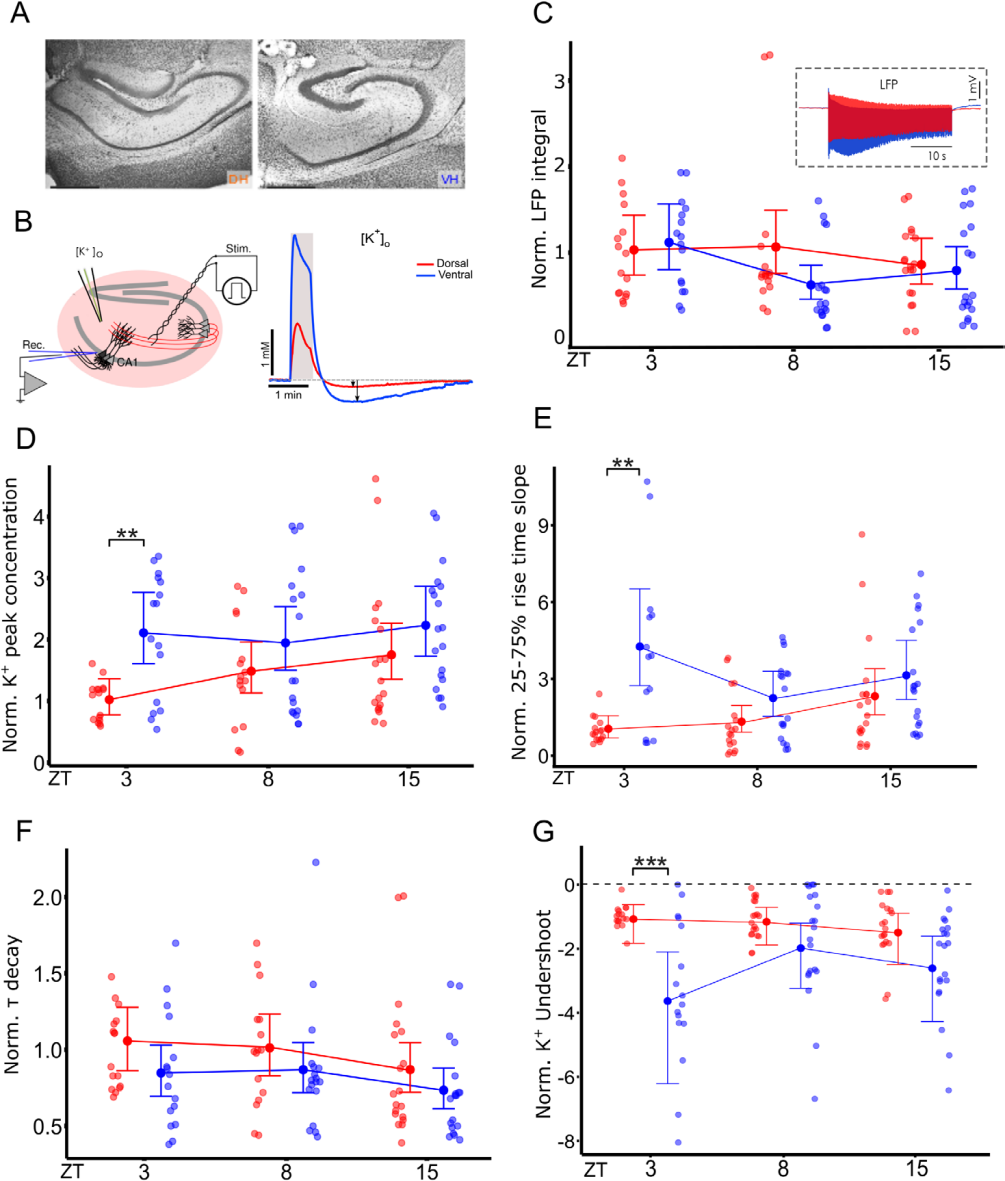
Network level extracellular K+ recording. (A) Example of anatomical structures of dorsal (DH: Right; red in plots) and ventral (VH: Left; blue in plot) hippocampal slices. (B) Schematic arrangement of stimulation of Schaffer Collateral (SC), recording of local field potential (LFP) from the *stratum oriens* (*s.o*.), and extracellular K+ [K^+^]_o_ recording from *stratum radiatum* (*s.r*.). Example trace of [K^+^]_o_ in DH (red) and VH (blue) in response to 10 Hz 30 s stimulation (the gray bar). (C–G) Normalized LFP integral, 25%–75% rise time, peak amplitude, tau decay of monoexponentially fitted falling phase of the K^+^ transient, and undershoot amplitude across DH and VH in three ZT3, ZT8, and ZT15. VH exhibits larger and faster extracellular K transients and larger post‐stimulus undershoot than DH, particularly at ZT3. Analyses, based on a lognormal distribution model, are reported as posterior median estimates with 95% credible intervals. ** and *** indicate that pairwise 95% Highest Posterior Density (HPD) credible intervals exclude zero. For legacy purposes, these markers also correspond to Bonferroni‐corrected frequentist values of *p* < 0.01 and *p* < 0.001, respectively. [DH_ZT3: *N* = 16 (*N* = 8), DH_ZT8: *N* = 16 (*N* = 8), DH_ZT5: *N* = 19 (*N* = 10), VH_ZT3: *N* = 16 (*N* = 8), VH_ZT8: *N* = 18 (*N* = 9), VH_ZT5: *N* = 19 (*N* = 10)]. As in the following, N indicates the number of animals while n indicates the number of slices.

We first characterized how extracellular K^+^ concentration ([K^+^]_o_) changes in the dorsal and ventral hippocampus as a function of time in response to network activity. Since synaptic transmission is modulated in a circadian manner (Snider et al. 2018), we adjusted the stimulus intensity to evoke similar field responses to enable comparisons across conditions: VH versus DH and ZT (Figure 1A,B). The 10 Hz, 30‐s stimulation of Schaffer collaterals protocol also ensured that the neuronal network response reached true peak amplitude and steady state before stimulus termination (see Methods).

The LFP integral, used as a proxy for network response intensity, was similar between regions and ZT (Figure 1C). Permutation ANOVA did not reveal effects of region, time, or their interaction on LFP integral, and Bayesian multilevel models yielded posterior intervals that included zero for all contrasts. Taken together, these analyses show that our protocol standardizes the network response across conditions. Thus, any subsequent differences in [K^+^]_o_ responses should reflect genuine changes in [K^+^]_o_ buffering dynamics.

#### 3.1.2 Ventral Hippocampus Accumulates [K^+^]_o_ More Rapidly and to Greater Amplitudes, Particularly at Early Light Phase

We first quantified the peak amplitude of the K^+^ transient as an index of extracellular K^+^ accumulation. Permutation ANOVA revealed a significant main effect of region (F1,98 = 12.05, permutation *p* = 0.0010, ω^2^ = 0.0950), with pooled VH samples showing a 65% larger K peak amplitude than DH (95% CI 26, 101; Figure 1D). This effect size was moderate, indicating a reliable dorsoventral difference.

Bayesian regression further indicated that this regional difference was most evident at ZT3. At this time point, K^+^ peak amplitude in VH was 105% higher than in DH (95% CrI 40.5, 200.4; Figure 1D). Within DH, the transition from ZT3 to ZT15 increased peak amplitude by 71.6% (95% CrI 17.4, 150.9), indicating that extracellular K^+^accumulation in dorsal slices also varied across circadian time.

We next analyzed the 25%–75% rise slope to assess the rate of K^+^ accumulation. At ZT3, VH reached peak K^+^ 305.5% faster than DH (95% CI 124.8, 646.3; Figure 1E) with a very large effect size. In DH, the change from ZT3 to ZT8 was not clearly different from 0 (27.1%, 95% CrI −26.7, 120.3), whereas the change from ZT3 to ZT15 was associated with a 122.6% increase in accumulation rate (95% CrI 28.4, 293.5). The specificity of K^+^ accumulation in VH at ZT3 was reduced at later times, with the VH effect attenuated by 41.7% at ZT8 (95% CrI 19.0, 93.1) and by 33.3% at ZT15 (95% CrI 14.9, 72.7), consistent with a negative region‐by‐time interaction (Figure 1E).

Together, these analyses indicate that, under comparable neuronal activation, extracellular K^+^ accumulates faster and to a greater level in VH than in DH, particularly at ZT3. The estimated differences are not only statistically supported but also large enough to be physiologically meaningful.

#### 3.1.3 [K^+^]_o_ Clearance Remains Broadly Similar Across Regions

To determine whether the larger K^+^ transients in VH were also associated with slower recovery, we quantified the decay phase of the transient by monoexponential fitting (Figure 1F). Permutation ANOVA detected a small main effect of region (F1,98 = 3.93, permutation *p* = 0.0496, ω^2^ = 0.0278), with VH showing an overall 14.5% faster decay than DH (95% CI −0.20, 28.1). The effect size was compatible with small to negligible effects, and the confidence interval nearly included 0, indicating limited support for a robust regional difference in decay kinetics.

Bayesian regression did not identify statistically robust time‐specific regional differences. At ZT3, DH decayed 22.3% more slowly than VH (95% CrI −5.9, 50.2); at ZT8, the difference was 15.2% (95% CrI −14, 41.5); and at ZT15, the difference was 17.0% (95% CrI −11, 42.1; Figure 1F). Because all credible intervals crossed 0, this finding should be regarded as analysis‐dependent and tentative rather than as a central regional effect.

Thus, the main regional difference lies in the rising phase of the K^+^ transient rather than in the decay phase. The combined frequentist and Bayesian analyses are most consistent with broadly similar K^+^ clearance rates across regions, suggesting that distinct buffering mechanisms may be preferentially engaged during accumulation versus recovery.

#### 3.1.4 Ventral Hippocampus Shows a Larger Post‐Stimulus Undershoot at ZT3

We next examined the post‐stimulus K^+^ undershoot (Figure 1B,G), which follows return to baseline after prolonged stimulation and is commonly used as a readout related to Na, K‐ATPase‐dependent recovery mechanisms (Chever et al. 2010; D'Ambrosio et al. 2002). Permutation ANOVA revealed a significant main effect of region (F1,105 = 26.65, permutation *p* = 0.0001, ω^2^ = 0.180) and a significant region‐by‐time interaction (F2,105 = 3.074, permutation *p* = 0.0482, ω^2^ = 0.0290; Figure 1G). The regional effect size was large, whereas the interaction effect size was small, indicating that the dominant feature of the data is an overall dorsoventral difference with a more limited temporal modulation.

Bayesian analysis showed that at ZT3, VH exhibited a 239% larger undershoot amplitude than DH (95% CrI 54, 624; Figure 1G). Although undershoot amplitude remained elevated in VH at ZT8 and ZT15, the magnitude of the regional difference was reduced, with interaction terms of −49% at ZT8 (95% CrI −82, 42) and −48% at ZT15 (95% CrI −81, 48). Because the credible intervals for these interaction terms crossed 0, the strongest support for an enhanced ventral undershoot was observed at ZT3.

Taken together, Figure 1 shows that VH exhibits larger and faster extracellular K transients than DH, particularly at ZT3, whereas decay kinetics are broadly similar and the post‐stimulus undershoot is larger in VH. The effect‐size pattern indicates that region contributes most strongly to K accumulation and undershoot amplitude, but only weakly to decay kinetics. Despite the putative elevated pump activity in VH, overall [K^+^]_o_ clearance rates remain unchanged, suggesting that the enhanced pump activity primarily reflects the greater [K^+^]_o_ accumulation in VH (which provides greater driving force for pump activation) rather than fundamentally altering the rate constant of [K^+^]_o_ removal. This raises the question: what cellular mechanisms limit [K^+^]_o_ buffering capacity in the ventral hippocampus?

### 3.2 Cellular Basis of Regional [K^+^]_o_ Buffering Differences: A Role for Reduced Kir4.1 Function in Ventral Astrocytes

#### 3.2.1 Astrocytic Gap‐Junction Coupling Shows Regional and Temporal Variation

**FIGURE 2:**
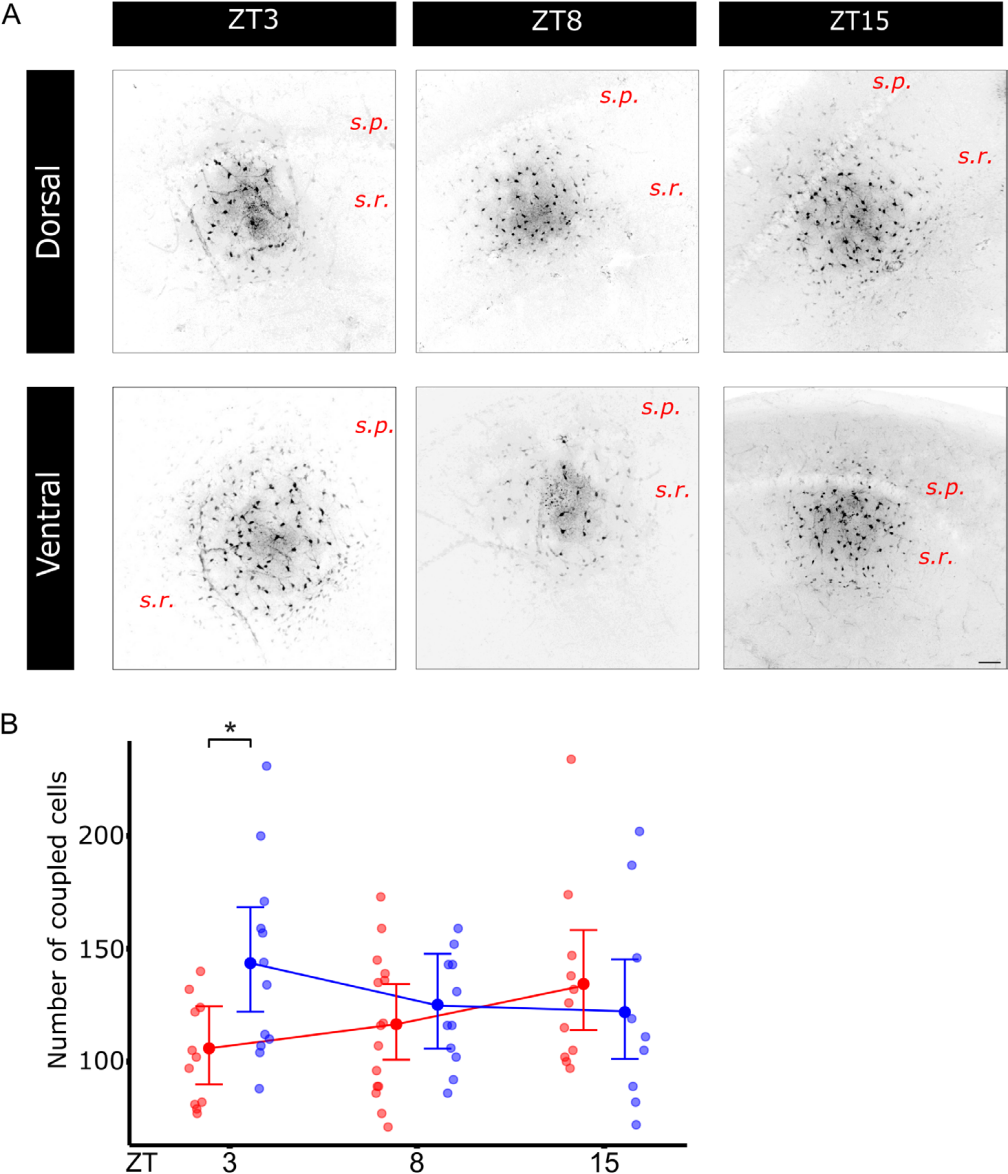
Gap junction coupling across hippocampal regions and time of day. (A) Inverted example image of the coupled astrocytes in a hippocampal slice, as obtained after biocytin diffusion from a single cell, held in whole‐cell voltage clamp for 20 min, across noted regions and ZTs. Abbreviations: *S.p.: Stratum Pyramidale, s.r.: Stratum Radiatum*. Scale bar: 50 μm. (B) Summary of the number of biocytin‐coupled cell across DH and VH at different time points (ZT3, 8, 15). Analyses, based on a lognormal distribution model, are reported as posterior median estimates with 95% credible intervals. * indicates that pairwise 95% Highest Posterior Density (HPD) credible intervals excludes zero. For legacy purposes, this marker corresponds to Bonferroni‐corrected *p* < 0.05 after pairwise post hoc analysis. [DH_ZT3: *N* = 11 (*N* = 6), DH_ZT8: *N* = 15 (*N* = 6), DH_ZT5: *N* = 11 (*N* = 6), VH_ZT3: *N* = 12 (*N* = 7), VH_ZT8: *N* = 11 (*N* = 7), VH_ZT5: *N* = 9 (*N* = 6)].

Potassium redistribution through astrocytic networks depends on gap junction‐mediated coupling, which expands the effective volume available for [K^+^]_o_ uptake. To assess whether gap‐junction‐mediated coupling varies across hippocampal region and time, coupling was estimated by quantifying the number of biocytin‐labeled cells connected to a single patched astrocyte (Figure 2A,B). Because this outcome has hierarchical structure and a non‐Gaussian distribution, the Bayesian multilevel model was treated as the primary analysis, whereas permutation ANOVA is reported as a complementary non‐parametric check.

Across all time points, VH showed a trend toward more coupled astrocytes than DH, with a mean difference of 13 cells (95% CI −4, 30; Figure 2B). At the omnibus level, this regional effect did not reach the conventional significance threshold in the permutation ANOVA (reported as F2,63 = 2.75, permutation *p* = 0.0686, ω^2^ = 0.0483). This effect size was small, and the confidence interval crossed 0, indicating limited support for a global regional effect when data were pooled across time.

Bayesian analysis resolved a time‐dependent effect. At ZT3, VH exhibited 39 more coupled cells than DH (95% CrI 15, 69), corresponding to a 36.3% increase (95% CrI 8.3, 70.0; Figure 2B). This regional difference was not evident at ZT8. At ZT15, coupling in DH increased by 27% relative to its ZT3 value (95% CrI 1.00, 60.0), whereas coupling in VH declined by 16.2% (95% CI 6.8, 52.3), indicating convergence between regions later in the cycle (Figure 2A,B).

Thus, astrocytic coupling varies with both region and time and is increased in VH at ZT3, the same condition in which extracellular K^+^ accumulation is maximal. However, because the omnibus effect is small and the support for a regional difference depends on the time‐resolved model, this finding is best interpreted as temporally specific rather than as a strong global dorsoventral effect. These results suggest that reduced K^+^ conductance mechanisms (rather than impaired redistribution) are the primary determinant of regional differences.

#### 3.2.2 Reduced Kir4.1‐Mediated Conductance in Ventral Versus Dorsal Hippocampal Astrocytes Across Circadian Phases

##### 3.2.2.1 Resting Membrane Potential Does Not Differ Across Region or Time

**FIGURE 3:**
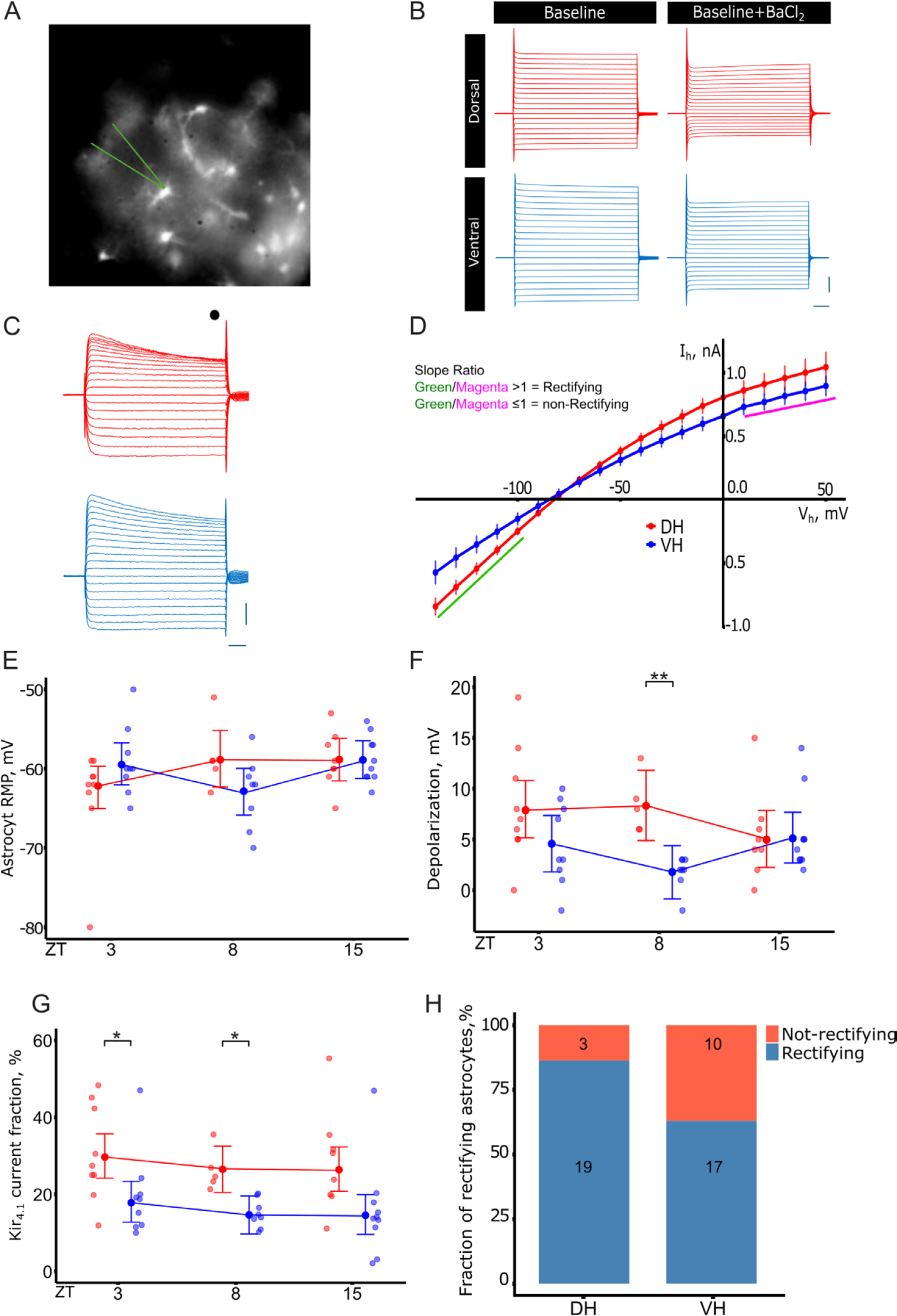
Regional differences across regions and time. (A) Wide‐field image of stained astrocytes with SR101 in the hippocampal *s.r*. (B) Example of current–voltage relationship of Dorsal (red) and Ventral (blue) hippocampal slices in baseline and BaCl_2_ ACSF. Scale bars; horizontal: 50 ms, vertical: 2 nA. (C) Traces of currents as a result of subtraction of Baseline from Ba^2+^ traces obtained from Dorsal (red) and ventral (blue) slices. The last 50 ms of the trace, noted by the black dot, was used to obtain the current–voltage curve. Scale bars; horizontal: 50 ms, vertical: 500 pA. (D) Example of current–voltage relationship of Ba^2+^‐sensitive currents measured in DH (red) and VH (blue) astrocytes. Ba^2+^‐sensitive currents were isolated by subtracting membrane currents recorded in the presence of Ba^2+^ from baseline whole‐cell currents (voltage steps from −140 mV to +50 mV in 10 mV increments; V_hold_ = −70 mV). To calculate the rectification profile of the tested cell, the slope of the I‐V relationship was measured at two distinct voltage intervals: The inward current slope between −140 mV and −100 mV (green) and the outward current slope between +100 mV and +50 mV (magenta). The rectification index was then determined by the ratio of these two slopes, characterizing the voltage‐dependent conductance properties of the astrocyte membrane. (E) Baseline resting membrane potential (RMP; in mV) of the patched astrocytes across DH and VH at ZT3, ZT8, and ZT15. (F) Ba^2+^‐induced depolarization across the regions and time. No significant effect of region and time was observed, except at ZT8. (G) Kir4.1 current across the regions and time of day. Significant main effect of region was observed without the influence of time. (H) Number of rectifying astrocytes (blue) versus non‐rectifying (orange) across the pooled samples of DH and VH. [DH_ZT3: *N* = 9 (*N* = 7), DH_ZT8: *N* = 5 (*N* = 3), DH_ZT5: *N* = 8 (*N* = 6), VH_ZT3: *N* = 9 (*N* = 6), VH_ZT8: *N* = 8 (*N* = 5), VH_ZT5: *N* = 10 (*N* = 5)] * and ** indicate that pairwise 95% HPD credible intervals exclude zero. For legacy purposes, these markers correspond to Bonferroni‐corrected *p* < 0.05 and *p* < 0.01, respectively, after pairwise post hoc analysis.

To test whether regional differences in K^+^ handling are associated with differences in Kir4.1‐mediated current (Larsen et al. 2014; Seifert et al. 2009), whole‐cell recordings were performed from CA1 *stratum radiatum* astrocytes under conditions designed to isolate the Ba^2+^‐sensitive current component (Wallraff et al. 2006) (Figure 3A–D). The uncoupling strategy used for Kir4.1 isolation was validated in Figure S1. MFA reduced the number of dye‐coupled cells by 71.4% on average (95% CI 60.0, 83.7; *p* < 0.00001; Figure S1B), and syncytium size was negatively correlated with input resistance (Spearman's rho = −0.502, *p* = 0.0089, *n* = 26; Figure S1C). These data confirm that MFA reduced both structural and functional coupling under the recording conditions used for Figure 3. For the following hierarchical cell‐based electrophysiological outcomes, Bayesian multilevel modeling was considered the primary inferential framework, with permutation ANOVA used as a robustness check. Resting membrane potential did not vary significantly between regions or across time (Figure 3E), indicating that baseline astrocyte membrane polarization was comparable across conditions.

##### 3.2.2.2 Ba^2+^‐Induced Depolarization Is Larger in DH Than in VH

Application of Ba^2+^ caused an average depolarization of 5.5 ± 0.64 mV consistent with blockade of inward‐rectifying currents, confirming effective channel blockade. Permutation ANOVA revealed a significant main effect of region on Ba^2+^‐induced depolarization (F(1, 43) = 8.26, permutation *p* = 0.0051, ω^2^ = 0.124), with DH astrocytes depolarizing 3.2 mV [95% CI: 0.90, 5.70 mV] more than VH astrocytes following Ba^2+^ application (Figure 3F). This effect size was moderate to large, indicating that region accounts for a meaningful fraction of the variance in this measure. The confidence interval did not include 0, supporting a larger Ba^2+^‐sensitive contribution to membrane potential in DH astrocytes.

##### 3.2.2.3 Ba^2+^‐Sensitive Kir4.1 Conductance Is Reduced in VH

We next quantified Ba^2+^‐sensitive current at −130 mV holding potential, a voltage optimal for Kir4.1 channel activity (Seifert et al. 2009). Permutation ANOVA revealed a significant effect of region (F(1, 43) = 13.30, permutation *p* = 0.0006, ω^2^ = 0.209).

Ba^2+^‐sensitive conductance represented 29% of total transmembrane current in DH astrocytes and 17% in VH astrocytes, corresponding to a mean difference of 12% (95% CI 5.8, 17.6; Figure 3G). Bayesian regression confirmed a main effect of region, with no detectable effect of time and no region‐by‐time interaction. Thus, the reduced Kir4.1‐mediated conductance in VH persisted across all circadian phases examined.

These data provide some of the strongest evidence in the study for a stable dorsoventral difference. The large effect size, together with interval estimates well separated from 0 and concordant Bayesian results, indicates that the regional difference in Kir4.1‐mediated conductance is both statistically robust and biologically substantial.

This reduced Kir4.1‐mediated conductance in VH astrocytes provides a mechanistic explanation for the observed rapid and pronounced K^+^ accumulation in this region: with lower K^+^ conductance, astrocytes can passively take up less K^+^ in response to depolarization, resulting in higher [K^+^]_o_ despite the presence of enhanced gap junction coupling that could support redistribution.

##### 3.2.2.4 Rectification Status Shows a Similar Trend but Remains Uncertain

Because some astrocytes displayed Ba^2+^‐sensitive current–voltage relationships that were more linear than rectifying, we compared the proportion of rectifying cells across regions (Figure 3H). Fisher's exact test did not detect a significant association between region and rectification status (*p* = 0.104), although the estimated odds ratio was 0.28 (95% CI 0.04, 1.31), suggesting a trend toward a greater proportion of rectifying cells in DH.

The confidence interval was broad and included 1.0, so the present data do not provide strong evidence for a categorical regional difference in rectification status.

#### 3.2.3 Kir4.1 Protein Expression Shows Region‐Dependent Circadian Regulation

**FIGURE 4:**
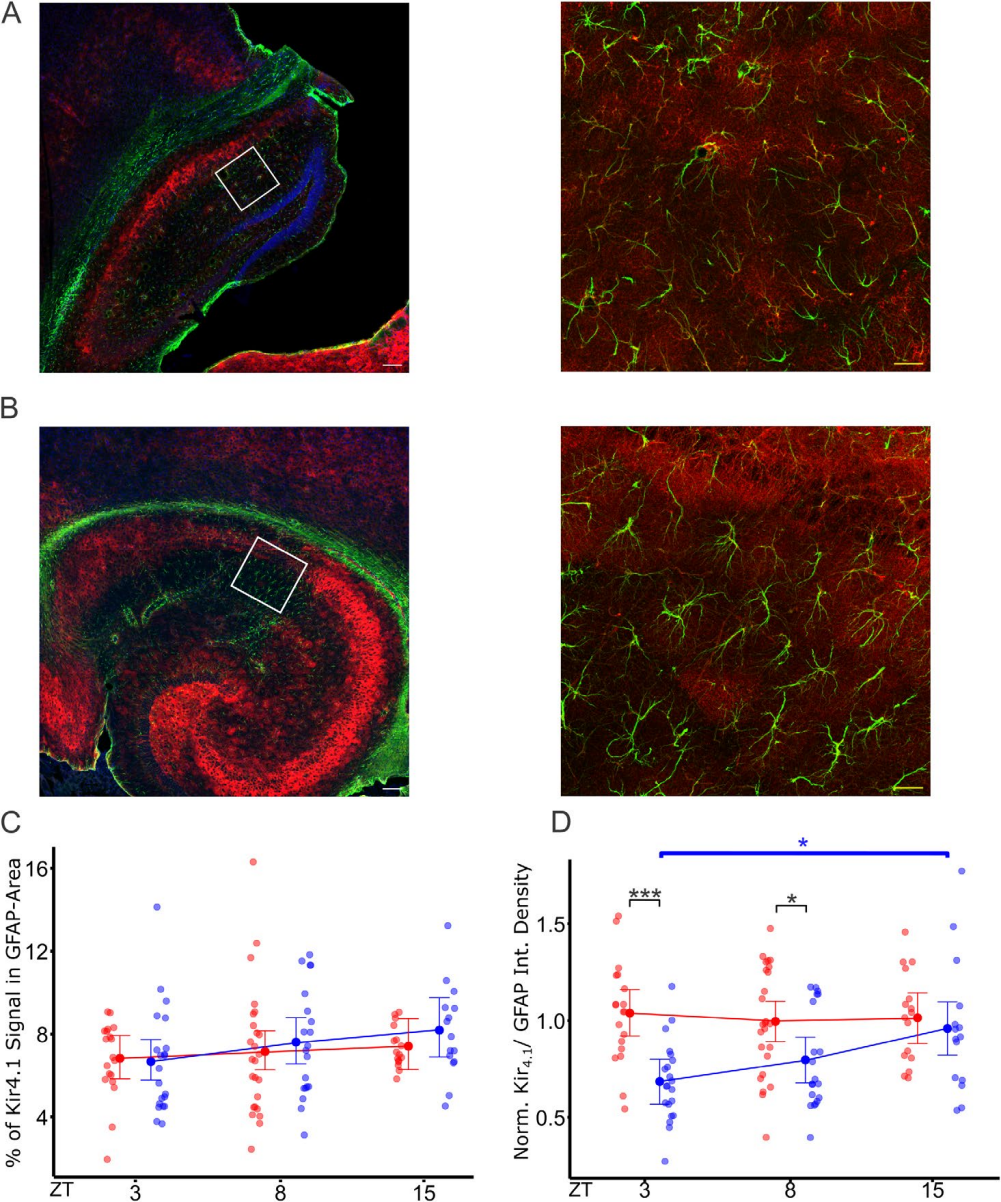
The regional differences in Kir4.1 expression along the dorsoventral axis depend on time. (A) Left: Representative example of confocal images of immunofluorescent labeling of Kir4.1 (red) and astrocyte marker glial fibrillary acidic protein (GFAP, green), and DAPI (blue) for DH, at 20× magnification in DH (scale bar = 100 μm). Right: Higher magnification of the 5 × 5 tile scan in the white square from the left image. (B) same as (A) for VH. (C) Fraction of GFAP positive area with Kir4.1 protein was comparable across regions and time. (D) Quantification of Kir4.1 integrated density normalized to GFAP. The difference was significant at ZT3, where VH showed 34.4% [95% CI −17.7%, 50.8%] less than DH. In VH, ZT15 showed 27.4% [95% CI 8.61%, 44.3%] more normalized integrated density as compared to ZT3, signifying significant interaction of region with time. Analyses, based on a student t‐distribution model, are reported as posterior median estimates with 95% credible intervals. * and *** indicate that pairwise 95% Highest Posterior Density (HPD) credible intervals exclude zero, respectively *p* < 0.05 and *p* < 0.001for legacy purposes after Bonferroni correction of *p*‐value [DH_ZT3: *N* = 19 (*N* = 4), DH_ZT8: *N* = 25 (*N* = 4), DH_ZT5: *N* = 17 (*N* = 4), VH_ZT3: *N* = 21 (*N* = 4), VH_ZT8: *N* = 20 (*N* = 4), VH_ZT5: *N* = 15 (*N* = 4)].

To determine whether the regional difference in Kir4.1 current was paralleled by a difference in protein expression, sections were co‐labeled for GFAP and Kir4.1 and quantified in CA1 *stratum radiatum* (Figures 4A,B, S2A–C). Because multiple images were nested within animals and staining batches, these image‐based data were interpreted with the same emphasis on effect size and uncertainty as the cell‐based outcomes.

Analysis of GFAP integrated density shows only the main effect of time (F(2, 110) = 3.15, permutation *p* = 0.0464, ω^2^ = 0.035), with Bayesian analysis indicating that this effect was limited to DH, where ZT15 was 24% higher than ZT3 (95% CI 3.5, 40.0; Figure S2D). The effect size was small, indicating that time explained only a limited proportion of the variance in GFAP signal. By contrast, analysis of the fraction of GFAP‐positive area containing Kir4.1 signal revealed no significant effect of region, time, or interaction (Figure 4C), indicating no clear difference in astrocytic occupancy across conditions. The small effect sizes and wide credible intervals observed in the integrated density model, combined with the lack of difference in masked area, suggest that the effect of time was limited to sub‐threshold intensity fluctuations rather than significant morphological or structural remodeling of the GFAP network.

When Kir4.1 signal was normalized to GFAP integrated density, permutation ANOVA revealed a significant effect of region (F(1, 110) = 16.15, permutation *p* = 0.0004, ω^2^ = 0.11), with DH showing 21.2% greater normalized Kir4.1 signal than VH overall (95% CI 11.0, 30.6; Figure 4D). This effect size was moderate, indicating a reliable regional difference, although smaller than that observed for functional Kir4.1 conductance.

Bayesian regression indicated that the regional difference was most evident at ZT3, when DH exceeded VH by 34.0% (95% CrI 20.5, 45.1; Figure 4D). In VH, the transition from ZT3 to ZT15 increased normalized Kir4.1 expression by 27.4% (95% CrI 8.61, 44.3), consistent with a region‐by‐time interaction. In contrast, DH maintained relatively stable Kir4.1 levels across time points.

Thus, astrocyte‐associated Kir4.1 protein expression differed across region and time, with the strongest dorsoventral difference observed at ZT3. The smaller effect size for protein expression than for Kir4.1‐mediated current indicates that the functional regional difference exceeds what would be expected from protein abundance alone.

### 3.3 Integrated Pattern Across Measures

Across the measures examined, the largest regional effects were observed for K^+^ undershoot amplitude (ω^2^ = 0.180; Figure 1G) and Kir4.1‐mediated conductance (ω^2^ = 0.209; Figure 3G). Moderate regional effects were observed for K^+^ peak amplitude (ω^2^ = 0.0950; Figure 1D), Ba^2+^‐induced depolarization (ω^2^ = 0.124; Figure 3F), and normalized Kir4.1 protein expression (ω^2^ = 0.11; Figure 4D). In contrast, K^+^ decay kinetics (ω^2^ = 0.0278; Figure 1F), GFAP integrated density (ω^2^ = 0.035; Figure S2D), and the omnibus coupling analysis (ω^2^ = 0.0483; Figure 2B) showed small effects.

Overall, these findings indicate that the strongest dorsoventral differences are observed in the accumulation phase of the extracellular K^+^ and in Kir4.1‐mediated astrocytic conductance, whereas differences in decay kinetics and global coupling estimates are comparatively limited. This effect‐size pattern is consistent across the complementary permutation and Bayesian analyses and helps distinguish the central results from findings that are more sensitive to analytic framework.

## 4 Discussion

### 4.1 Integration and Mechanistic Synthesis

This study addresses the question of spatial and temporal heterogeneity in astrocytic function. Using integrated functional, electrophysiological, and molecular approaches, we demonstrate that astrocytic K^+^ buffering in the hippocampus is jointly regulated by regional identity and circadian phase, with important implications for understanding network excitability, seizure susceptibility, and the temporal organization of brain states.

**FIGURE 5:**
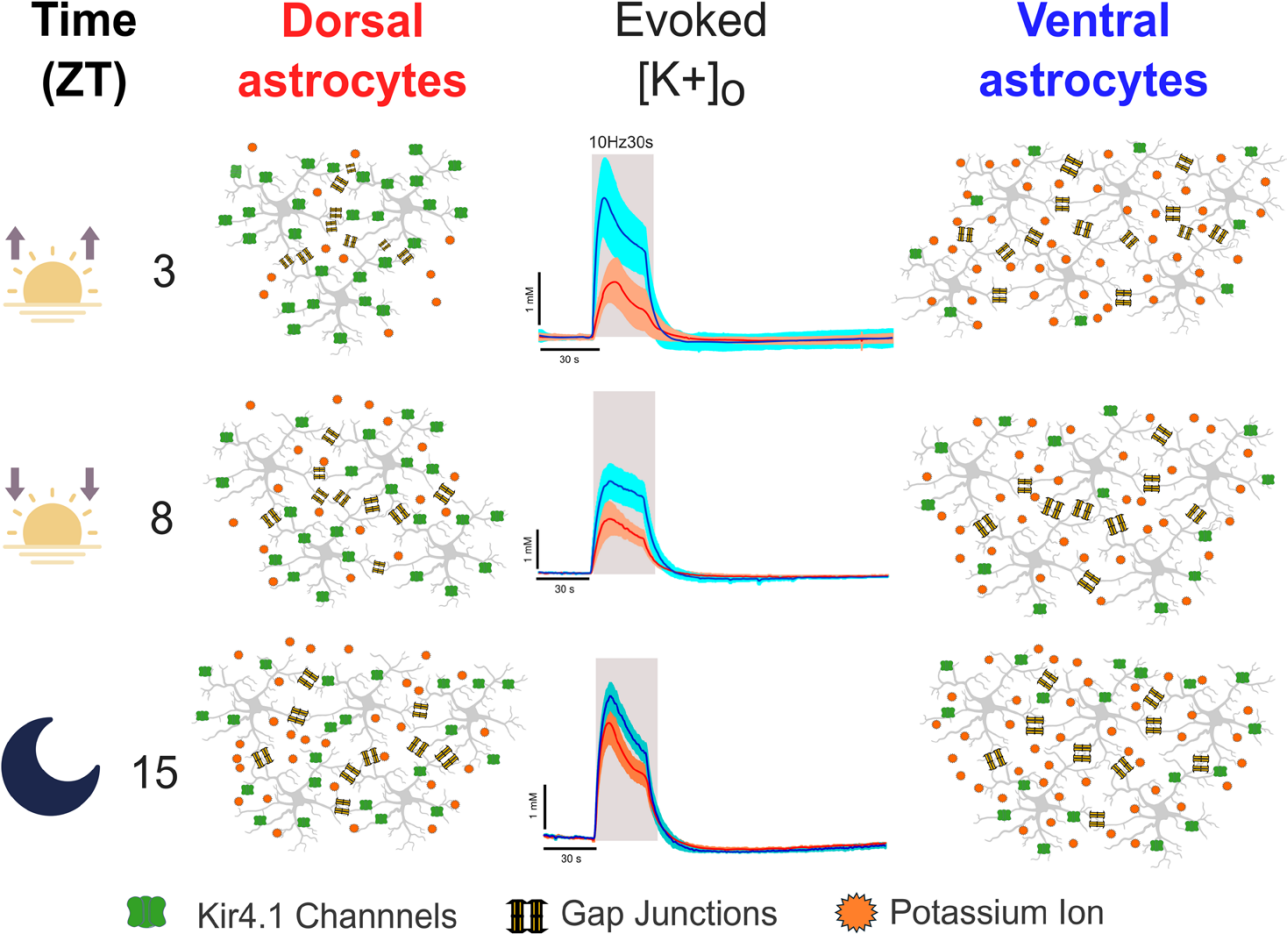
Graphical summary of the results. At the early light phase (ZT3), ventral hippocampus (VH) astrocytes exhibit rapid and pronounced [K^+^]_o_ accumulation during neuronal activity. This accumulation is driven by a persistent reduction in Kir4.1‐mediated conductance, limiting uptake during the rising phase despite compensatory increases in gap junction coupling and Na^+^/K^+^ ATPase pump activity. As the circadian cycle progresses to the early dark phase (ZT15), regional disparities attenuate: Kir4.1 protein expression in the VH increases to match the DH, while gap junction coupling and compensatory pump activity normalize. Conversely, [K^+^]_o_ accumulation in the DH increases by ZT15, suggesting a time‐dependent suppression of dorsal astrocytic buffering capacity during the light phase that is subsequently restored in the dark phase.

Specifically, our results reveal a multiscale organizational principle for this regulation: buffering capacity depends upon regional and circadian factors through complementary mechanisms operating at different phases of [K^+^]_o_ transients (Figure 5). At the early light phase (ZT3), the ventral hippocampus exhibits a state of heightened extracellular K^+^ accumulation characterized by: (1) reduced Kir4.1‐mediated K^+^ conductance (41% lower than DH) that persists across all times, (2) enhanced gap junction coupling (35% more coupled astrocytes) that could support redistribution, and (3) an elevated Na^+^/K^+^‐ATPase‐dependent undershoot (239% greater than DH) suggesting compensatory pump activation. These properties produce rapid and pronounced [K^+^]_o_ accumulation despite the presence of potentially advantageous gap junction coupling. The putative enhanced Na^+^/K^+^‐ATPase activity likely reflects activation by the greater local [K^+^]_o_ accumulation and astrocytic depolarization, coupled with potentially higher pump expression. Thus, at ZT3, ventral astrocytes are limited in their capacity to prevent [K^+^]_o_ accumulation during the rising phase.

As the circadian cycle progresses through the light to dark phases (ZT3 to ZT8 to ZT15): (1) Kir4.1 protein expression increases in the VH while it remains stable in the DH, eventually equalizing regional differences by ZT15; (2) gap junction coupling normalizes between regions; and (3) Na^+^/K^+^‐ATPase compensatory activity diminishes.

By the early dark phase (ZT15), the circadian pattern reverses, with DH [K^+^]_o_ accumulation increasing relative to ZT3, suggesting that dorsal hippocampal astrocytes are subject to time‐dependent suppression of buffering capacity during the light phase, with restoration during the dark phase. The nature of this circadian suppression remains to be determined but may reflect circadian regulation of Kir4.1 trafficking or channel modulation by clock‐controlled signaling pathways.

### 4.2 Astrocytes Show Regional Specialization That Do Not Simply Mirror Neuronal Excitability Patterns

Our findings reveal that ventral hippocampal astrocytes possess different [K^+^]_o_ buffering properties as compared to dorsal astrocytes. At the early light phase (ZT3), when regional differences are most pronounced, VH accumulates [K^+^]_o_ more rapidly and to higher peak amplitudes than DH despite equivalent neuronal network activity. This regional divergence reflects intrinsic differences in astrocyte physiology rather than variations in neuronal input, establishing that astrocytic heterogeneity is a primary organizational principle of hippocampal circuits.

However, astrocytic regional specialization does not simply track neuronal excitability in a compensatory manner. The VH is known to exhibit higher intrinsic neuronal excitability and greater seizure susceptibility than the DH (Debski et al. 2020; Dougherty et al. 2012; Papatheodoropoulos et al. 2005). If astrocytes were optimized to compensate for regional differences in neuronal activity, one would predict enhanced [K^+^]_o_ buffering capacity in VH to counterbalance its elevated excitability. Instead, we observe the opposite: VH astrocytes display reduced Kir4.1‐mediated conductance (12% lower than DH), resulting in greater [K^+^]_o_ accumulation during neuronal activity. This counterintuitive arrangement suggests that astrocytic properties are not configured to suppress regional differences in excitability but rather may amplify or enable them, supporting the functionally distinct roles mediated by the dorsal and ventral hippocampus in cognition and behavior.

### 4.3 The Paradox of Enhanced Accumulation With Preserved Clearance: Differential Mechanisms at Rising Versus Falling Phases

A central finding of this study is the paradoxical observation that VH exhibits both faster [K^+^]_o_ accumulation and similar (or slightly faster) clearance rates as compared to DH. This apparent contradiction is resolved by recognizing that distinct buffering mechanisms dominate during different phases of the K^+^ transient, with Kir4.1 channels playing a central role during accumulation and Na^+^/K^+^‐ATPase predominating during recovery.

In this study, Na^+^/K^+^ ATPase activity was quantified using the amplitude of the post‐stimulus K^+^ undershoot rather than by pharmacological pump inhibition because it does not selectively reduce [K^+^]_o_ clearance. Indeed, the pump inhibition, via application of ouabain for example, depolarizes neurons, alters firing patterns, and modifies synaptic transmission (T. R. Anderson et al. 2010), thereby changing K+ release during Schaffer collateral stimulation. Consequently, [K^+^]_o_ transients recorded after pump blockade reflect a mixture of impaired clearance and altered K^+^ release, which cannot be disentangled. In contrast, the [K^+^]_o_ undershoot that follows a defined activation epoch arises from activity‐dependent stimulation of the Na^+^/K^+^ ATPase and reflects the excess pump‐mediated uptake of K^+^ once neuronal firing has ceased. For these reasons, undershoot analysis offers a more specific and interpretable functional measure of Na^+^/K^+^ ATPase activity in our experimental condition.

During the rising phase of neuronal activity, when K^+^ is actively released from depolarizing neurons, [K^+^]_o_ elevation depolarizes nearby astrocytes, driving passive K^+^ influx through Kir4.1 channels down the electrochemical gradient (Larsen and MacAulay 2014). This Kir4.1‐mediated uptake represents the fastest way to limit [K^+^]_o_ accumulation. Our electrophysiological data demonstrate that VH astrocytes possess significantly reduced Ba^2+^‐sensitive (Kir4.1‐mediated) conductance compared to DH astrocytes, a decrease that persists across all circadian phases examined. This reduced conductance directly explains why VH accumulates [K^+^]_o_ more rapidly: with fewer functional Kir4.1 channels, astrocytes cannot buffer K^+^ as efficiently during the rising phase, resulting in higher extracellular concentrations.

In contrast, during the decay phase after neuronal activity ceases, [K^+^]_o_ clearance is dominated by active Na^+^/K^+^‐ATPase pumping rather than passive Kir4.1‐mediated influx (D'Ambrosio et al. 2002; MacAulay 2020). Our observation that VH exhibits an enhanced [K^+^]_o_ undershoot (239% larger than DH at ZT3)—a signature of robust Na^+^/K^+^‐ATPase activity—indicates that pump‐mediated clearance is elevated in VH. Importantly, this enhanced pump activity likely reflects a compensatory response to the greater [K^+^]_o_ accumulation itself: higher [K^+^]_o_ and greater astrocytic depolarization both stimulate Na^+^/K^+^‐ATPase activity (Wang et al. 2012). Thus, the similar decay kinetics between regions emerge not from equivalent intrinsic clearance capacity but from activity‐dependent upregulation of pumping in VH that compensates for its reduced Kir4.1 function.

This mechanistic model—reduced Kir4.1‐mediated uptake during accumulation, compensated by enhanced pump‐mediated clearance during recovery—reconciles the paradoxical K^+^ dynamics and highlights the phase‐specific contributions of different buffering mechanisms.

### 4.4 Gap Junction Coupling: Enhanced Connectivity Cannot Overcome Reduced Conductance

An unexpected finding was that VH astrocytes exhibit enhanced gap junction coupling at ZT3 (35% more coupled cells than DH), precisely when and where K^+^ accumulation is greatest. At first glance, this enhanced coupling appears maladaptive: expanded astrocytic networks should increase the effective volume available for K^+^ redistribution and thus improve buffering capacity (Wallraff et al. 2006); yet VH accumulates more [K^+^]_o_.

This paradox highlights an important distinction between network connectivity (assessed by gap junction coupling) and cellular K^+^ conductance (determined primarily by Kir4.1 expression). Enhanced coupling expands the astrocytic syncytium, but K^+^ must first enter astrocytes through Kir4.1 channels before it can be redistributed through the network. With Kir4.1 conductance reduced by 12% in VH astrocytes, the membrane represents a bottleneck that limits K^+^ uptake regardless of how extensive the downstream network may be. Thus, gap junction coupling appears to represent a partial compensatory adaptation—expanding the potential redistribution capacity—but this adaptation cannot fully overcome the primary reduction (as compared to DH) in membrane conductance.

This suggests that Kir4.1 channel function, rather than gap junction coupling, is the rate‐limiting step for K^+^ buffering during active neuronal stimulation in VH.

### 4.5 Circadian Regulation Superimposed on Stable Regional Identity

Two key parameters exhibit no circadian variation: (1) the time constant of K^+^ decay, which remains similar between regions and constant across time, and (2) Ba^2+^‐sensitive Kir4.1 current, which shows consistent regional differences (lower in VH) at all timepoints despite circadian changes in Kir4.1 protein expression.

The stability of Kir4.1 current despite changes in protein expression points to complex post‐translational regulation. At ZT3, DH exhibits 34% higher Kir4.1 protein expression than VH; by ZT8, this regional difference diminishes due to increased expression in VH. Yet functional Kir4.1 currents remain regionally differentiated throughout the day. This dissociation suggests that Kir4.1 channel activity is regulated not only by protein abundance but also by trafficking to the membrane, phosphorylation state, interaction with scaffolding proteins, or lipid microenvironment—mechanisms that may themselves be under circadian control and differ between regions.

The most pronounced circadian effect is observed in the convergence of multiple parameters at ZT3 versus later timepoints. At ZT3, the regional divergence between DH and VH is maximal: K^+^ peak amplitude differs 2.1‐fold, gap junction coupling differs by 35%, and Kir4.1 protein expression differs by 41%. By ZT8 and ZT15, these differences attenuate or reverse. This temporal pattern suggests that the early light phase—corresponding to the rest period in nocturnal rodents—may be a time when regional specialization is most important, possibly reflecting distinct roles for dorsal and ventral hippocampus in sleep‐dependent memory consolidation, sharp‐wave ripple generation, or metabolic recovery (Pronier et al. 2023).

### 4.6 Functional Implications: Regional K^+^ Dynamics Shape Circuit Computations

The distinct K^+^ buffering properties of DH and VH astrocytes have important implications for how these regions process information and contribute to hippocampal function. Extracellular K^+^ accumulation exerts multiple effects on neuronal excitability, synaptic transmission, and network oscillations, making astrocytic control of [K^+^]_o_ a powerful regulator of circuit dynamics.

On one hand, in the ventral hippocampus, K^+^ accumulation enables synchronized network events but imposes low‐pass filtering. The rapid and pronounced [K^+^]_o_ accumulation in VH has both facilitatory and inhibitory consequences. Elevated [K^+^]_o_ depolarizes neurons toward action potential threshold, enhancing NMDA receptor activation by relieving Mg^2+^ block and increasing the spatial range of glutamate spillover (Shih et al. 2013). These effects promote network synchronization and may facilitate the generation of hippocampal sharp‐wave ripples (SWRs)—brief, high‐frequency oscillations essential for memory consolidation. Indeed, VH slices more readily generate spontaneous SWRs than DH slices (Schlingloff et al. 2014), and CA3 pyramidal neurons in VH possess stronger recurrent connectivity and higher intrinsic excitability (Sun et al. 2020), properties that synergize with permissive K^+^ accumulation to support synchronized discharges.

However, sustained K^+^ elevation during prolonged activity also inactivates voltage‐gated Na^+^ channels and reduces the driving force for K^+^ efflux through voltage‐gated K^+^ channels, leading to synaptic depression and reduced responsiveness to repetitive stimulation (Meeks and Mennerick 2004). This creates a functional low‐pass filter, where VH responds robustly to initial or low‐frequency inputs but saturates during sustained high‐frequency activity. Such filtering may prevent runaway excitation and protect against seizure propagation, while simultaneously biasing VH toward processing sparse, salient events rather than sustained information streams.

In the dorsal hippocampus, on the other hand, tight K^+^ control preserves high‐fidelity transmission and supports frequency‐dependent facilitation. In contrast to VH, the strong Kir4.1‐mediated buffering in DH maintains lower [K^+^]_o_ during neuronal activity, preserving neuronal excitability and enabling synaptic facilitation at frequencies between 10 and 50 Hz—precisely the range that encodes behaviorally relevant information (Papaleonidopoulos et al. 2017). Lower K^+^ accumulation prevents synaptic depression, supporting sustained information transfer and communication with neocortical targets (Moreno et al. 2016). This configuration suits DH for high‐pass filtering: reliable transmission of sustained, structured activity patterns required for spatial navigation, episodic memory encoding, and cognitive map formation (Fanselow and Dong 2010).

The metabolic consequences of these regional differences are consistent with recent findings that VH relies more heavily on aerobic glycolysis—generating cytosolic ATP rapidly but inefficiently—compared to the oxidative phosphorylation favored in DH (Brancati et al. 2021). The elevated Na^+^/K^+^‐ATPase activity required to clear excess K^+^ in VH imposes substantial energetic demands that may be preferentially met by glycolytic metabolism.

### 4.7 Pathological Implications: Regional K^+^ Dysregulation and Epilepsy

The reduced K^+^ buffering capacity of VH astrocytes provides a mechanistic framework for understanding the well‐established vulnerability of VH to seizures. Evidence from human temporal lobe epilepsy (TLE) and animal models identifies VH as the primary seizure initiation zone (Babb et al. 1984; Bernasconi et al. 2003; Buckmaster et al. 2022; Toyoda et al. 2013). Our findings suggest that intrinsic differences in astrocytic K^+^ buffering—present even in healthy tissue—predispose VH to hyperexcitability.

During pathological conditions such as epilepsy, when neuronal activity is abnormally intense and synchronized, the limited Kir4.1 capacity in VH would result in excessive [K^+^]_o_ accumulation, reaching levels (> 10 mM) known to be associated with or even trigger seizures (de Curtis et al. 2018; Enger et al. 2015). Furthermore, epileptic tissue exhibits downregulation of Kir4.1 expression and function (Bataveljic et al. 2024; Romanos et al. 2020), exacerbating the baseline deficit in VH and creating a positive feedback loop, that is, reduced buffering results in greater K^+^ accumulation, which enhances excitability and thus more intense neuronal firing, favoring further K^+^ release.

The circadian modulation of K^+^ buffering we observe also provides a potential mechanism for the circadian rhythmicity of seizure occurrence documented in epilepsy patients and animal models (Chang, Leite, et al. 2018; Proix et al. 2021; Quigg et al. 2000). Seizure susceptibility peaks during specific circadian phases, and our data suggest that times of maximal regional divergence in K^+^ buffering (e.g., ZT3) may correspond to windows of heightened vulnerability in VH. Conversely, circadian phases where regional differences attenuate may represent periods of reduced seizure risk. However, given the extensive reorganization of circuits in epilepsy, it will be important to assess all the above‐described properties in experimental epilepsy.

### 4.8 Dissociation Between Kir4.1 Protein Expression and Functional Current: Post‐Translational Complexity

One of the most intriguing findings is the dissociation between Kir4.1 protein expression dynamics (which vary significantly across circadian time in VH) and functional Kir4.1‐mediated currents (which show stable regional differences but no temporal variation). This dissociation implies that channel function is regulated at multiple levels beyond simple protein abundance.

Several post‐translational mechanisms could account for this complexity. Kir4.1 channels must be trafficked to the plasma membrane to be functional, and this trafficking is regulated by interactions with scaffolding proteins such as dystrophin‐associated protein complex and α‐syntrophin (reviewed in (Olsen and Sontheimer 2008)). Circadian changes in trafficking machinery or cytoskeletal organization could alter the proportion of synthesized channels that reach the membrane. Additionally, Kir4.1 activity is modulated by phosphorylation, pH, and membrane lipid composition (Hibino et al. 2010)—all of which exhibit circadian oscillations (McCauley et al. 2020; Ryu et al. 2024). Finally, Kir4.1 channels interact with other membrane proteins in macromolecular complexes, and changes in these interactions could alter channel open probability or conductance without changing total protein levels.

The persistence of regional differences in functional current despite convergence of protein expression also suggests that DH and VH astrocytes may differ in their complement of regulatory proteins or post‐translational modifications. Identifying these region‐specific regulatory mechanisms represents an important direction for future research and could reveal novel targets for modulating astrocytic K^+^ buffering in disease states.

### 4.9 Comparative Context: Astrocytic Heterogeneity as a General Principle

Our findings contribute to a growing recognition that astrocytic heterogeneity is a fundamental organizational principle across the brain. Astrocytes differ between brain regions in molecular marker expression, morphology, calcium signaling dynamics, glutamate uptake capacity, and contribution to synaptic function (reviewed in (Khakh and Deneen 2019)). Within the hippocampus, dorsoventral differences in astrocytic gene expression, morphology, and calcium dynamics have been documented (Dong et al. 2009; Jinno 2011; Ryu et al. 2024; Thompson et al. 2008), but the functional consequences of this heterogeneity for circuit physiology have remained unclear.

Our demonstration that astrocytic K^+^ buffering—a core homeostatic function—exhibits pronounced regional and temporal variation establishes that astrocytic heterogeneity is not merely a descriptive phenomenon but has direct, measurable impacts on network excitability and information processing. This principle likely extends beyond the hippocampus to other brain structures where astrocytic properties may be tuned to local circuit demands.

Importantly, our findings challenge the traditional view of astrocytes as passive, uniform support cells that maintain brain homeostasis in a spatially and temporally invariant manner. Instead, astrocytes emerge as active, heterogeneous participants in circuit function whose properties are dynamically regulated in space and time. This has implications for understanding both normal brain function and the pathophysiology of neurological disorders.

### 4.10 Reconciling Slice Physiology With in Vivo Function: Caveats and Considerations

Several important caveats limit the interpretation of our findings. First, our measurements were performed in acute brain slices, which lack the intact vascular system, interstitial fluid flow, and long‐range connectivity present in vivo. K^+^ clearance mechanisms that rely on vascular uptake or long‐distance redistribution through white matter tracts may be compromised in slices, potentially altering the relative contributions of different buffering mechanisms. Evidence suggests that gap junction‐mediated spatial buffering contributes more to K^+^ clearance in vivo than in slices, raising the possibility that the enhanced gap junction coupling we observe in VH may be more functionally significant in the intact brain than our slice experiments suggest (Breithausen et al. 2020; Cooper et al. 2026).

While directly blocking gap junctions during stimulation would theoretically be the ideal way to test our bottleneck hypothesis, such an experiment is highly confounded in practice due to the off‐target effects of available gap junction inhibitors. To accurately assess astrocytic K^+^ buffering using evoked extracellular K^+^ transients, the baseline neuronal release of K^+^ must remain constant. However, pharmacological uncouplers directly alter neuronal network activity and excitability. For example, 100 μM MFA induces a rapid rundown and failure of sustained responses during train stimulation (Bojovic et al. 2025). Further tests on the inducible knock‐out of gap junction proteins may provide more insight on the effect of gap junction coupling on K^+^ dynamics (Hösli et al. 2022).

Second, the patch‐clamp approach in astrocytes samples a small radius of somata (Zhou et al. 2021), which may not fully represent the properties of fine perisynaptic processes where K^+^ buffering most directly impacts synaptic function. Recent advances in voltage imaging reveal that distal astrocytic processes experience larger depolarizations in response to neuronal activity than somata do (Armbruster et al. 2022), and spatial compartmentalization of ion channels may create local K^+^ buffering microdomains that our somatic recordings cannot detect. Additionally, we used GFAP immunolabeling to define astrocytic regions for Kir4.1 quantification, but GFAP labels only ~15% of astrocyte volume (Bushong et al. 2002), biasing our analysis toward the most GFAP‐rich cellular compartments and underestimating Kir4.1 in fine processes.

Third, our sampling of three circadian timepoints (ZT3, ZT8, ZT15) provides valuable initial insight into temporal regulation but may miss peak effects occurring at other phases. Higher temporal resolution studies examining more timepoints throughout the 24‐h cycle would provide a more complete picture of circadian dynamics.

Despite these limitations, our findings reveal fundamental principles of astrocytic K^+^ regulation that likely persist—and may be amplified—in vivo. The regional differences we document are substantial (2‐fold differences in K^+^ peak amplitude, 41% difference in Kir4.1 current) and consistent across multiple experimental approaches, suggesting they reflect robust biological properties rather than experimental artifacts.

Regarding the statistical analysis, the consideration of combined frequentist–Bayesian strategy was intended to increase transparency about the robustness of our conclusions to analytic choices, in line with recent proposals for multiverse and robust‐evidence analyses, rather than to “select” a single favored method or threshold, contributing to honest representation and subsequent interpretation of the data.

### 4.11 Future Directions and Therapeutic Implications

Our findings open several important avenues for future investigation. First, determining the molecular mechanisms underlying circadian regulation of Kir4.1 function—including identification of post‐translational modifications, trafficking pathways, and regulatory proteins that differ between DH and VH—could reveal novel targets for modulating astrocytic K^+^ buffering. Second, extending these studies to in vivo preparations using genetically encoded K^+^ sensors and astrocyte‐specific manipulations would clarify how the slice‐based findings translate to intact circuits and behavior. Third, investigating whether similar regional and temporal heterogeneity exists in the human hippocampus and whether these properties are altered in epilepsy could open the way to novel therapeutic avenues.

From a therapeutic perspective, our findings suggest that strategies to enhance astrocytic K^+^ buffering might need to be both regionally and temporally targeted. Global upregulation of Kir4.1 expression throughout the brain could have unintended consequences in regions where current expression levels are functionally appropriate. Similarly, chronotherapeutic approaches that time interventions to periods of maximum vulnerability—when regional differences in buffering capacity are most pronounced—may prove more effective than continuous treatment.

Gene therapy approaches to selectively enhance Kir4.1 expression in VH astrocytes represent a particularly promising avenue. Adeno‐associated viral vectors with astrocyte‐specific promoters (Tyurikova et al. 2025) could deliver Kir4.1 cDNA to VH, potentially normalizing its K^+^ buffering capacity to DH‐like levels and reducing seizure susceptibility. Alternatively, small molecules that enhance Kir4.1 channel open probability or trafficking to the membrane could achieve similar therapeutic effects through pharmacological means.

## 5 Conclusion

This study establishes that astrocytic K^+^ buffering in the hippocampus is neither spatially uniform nor temporally static but instead exhibits regional and circadian heterogeneity. Ventral hippocampal astrocytes possess intrinsically reduced Kir4.1‐mediated K^+^ conductance, resulting in rapid K^+^ accumulation during neuronal activity that is partially compensated by putative enhanced Na^+^/K^+^‐ATPase activity and expanded gap junction coupling. These regional specializations do not simply mirror neuronal excitability patterns but may actively shape them, creating distinct computational regimes in dorsal versus ventral hippocampus. Superimposed on this stable regional identity is a circadian layer of regulation that modulates—but does not abolish—regional differences, with maximal divergence during early light phase.

Our findings support the view that astrocytes are active, heterogeneous participants in circuit function whose homeostatic properties are dynamically tuned in space and time. This has implications extending beyond the hippocampus to other brain regions and beyond K^+^ buffering to other astrocytic functions. By revealing how regional and temporal factors converge to determine astrocytic K^+^ regulation, this work provides a mechanistic framework for understanding hippocampal circuit dynamics in health and offers potentially new therapeutic strategies for epilepsy based on targeted modulation of astrocytic function at specific times and locations.

## Author Contributions

N.K. and K.A. contributed equally to this work as first authors. N.K. performed patch‐clamp recordings, immunohistochemistry, and data and statistical analyses. K.A. performed field and extracellular K^+^ recordings, K^+^ feature extraction, and data post‐processing. A.I. co‐conceived the project, produced K^+^‐sensing electrodes, delineated brain slice regions, and analyzed K^+^ transient and field potential results. T.L.P. performed the blind counting of coupled cells and contributed to immunohistochemistry and microscopy. M.E. supervised microscopy and immunohistochemistry, provided secondary antibodies, and assisted in interpreting microscopy results. C.B. co‐conceived and supervised the overall project. N.K. drafted the initial manuscript, while C.B. and A.I. supervised the project and performed final editing. N.K., A.I., and C.B. interpreted the overall results and addressed reviewers’ comments. All authors contributed to the methods section and reviewed the manuscript.

## Funding

This work was supported by European Union's Horizon 2020 Research and Innovation Program MSCA‐ITN‐2020‐ASTROTECH(GA956325), Fondation pour la Recherche Médicale (FDT202404018111), Hrvatska Zaklada za Znanost (IP‐2022‐10‐8493), Petroleum Technology Development Fund (PTDF/ED/OSS/PHD/KA/2015/22).

## Conflicts of Interest

The authors declare no conflicts of interest.

## Supporting information


**Figure S1:** Effect of MFA on dye and electrical coupling. (A) Left: example image of biocytin stained astrocytes in absence of MFA (No MFA) versus incubated in MFA (right). *S.r., stratum radiatum*; *s.l.m., stratum lacunosum moleculare* (scale bar = 20 μm). (B) Statistical summary of the coupled cell number in both groups. MFA reduces the number of stained astrocytes. Addition of MFA results in an average reduction of 71.4% [95% CI 60.0%, 83.7%], *p* < 0.00001 calculated for legacy purposes only. (C) Scatter plot illustrating the relationship between the physical size of the syncytium (number of biocytin‐coupled cells) and passive membrane input resistance (Rin) following gap junction blockade with MFA. A robust, statistically significant negative correlation is observed (Spearman's *ρ* = −0.502, *p* = 0.0089, *n* = 26). The inverse relationship between coupling size and input resistance (R_in_) demonstrates that MFA‐induced reduction in dye transfer is accompanied by a concomitant decrease in electrical coupling.

**Figure S2:** Immunohistochemistry across times and regions. (A–C) Examples of confocal images of Kir4.1 (red) and astrocyte marker glial fibrillary acidic protein (GFAP, green), and the merging of the two images in the Stratum Radiatum of dorsal and ventral hippocampi across ZT3, ZT8, and ZT15, respectively. (D) Quantification of log_10_‐transformed GFAP integrated density across regions (dorsal hippocampus: red vs. ventral hippocampus: blue) and circadian times (ZT: 3, 8, 15). Only significant association was seen in the dorsal hippocampus where ZT15 shows 23.5% [95% CI 3.2, 39.2] more integrated density than at ZT3.

## Data Availability

The raw electrophysiology data, processed master datasets, and R analysis scripts that support the findings of this study are available on GitHub (https://github.com/Nari‐KIANI/Glia_3740732_Data) and permanently archived on Zenodo (https://doi.org/10.5281/zenodo.20933397).
