# Supplementary figures and images for: Dorso‐Ventral and Night‐Day Regulation of Extracellular K+ Dynamics in Mouse Hippocampal Astrocytes
Source: Glia. 2026 Jul 9;74(9):e70201. doi: 10.1002/glia.70201 (PMC13347764; doi:10.1002/glia.70201)

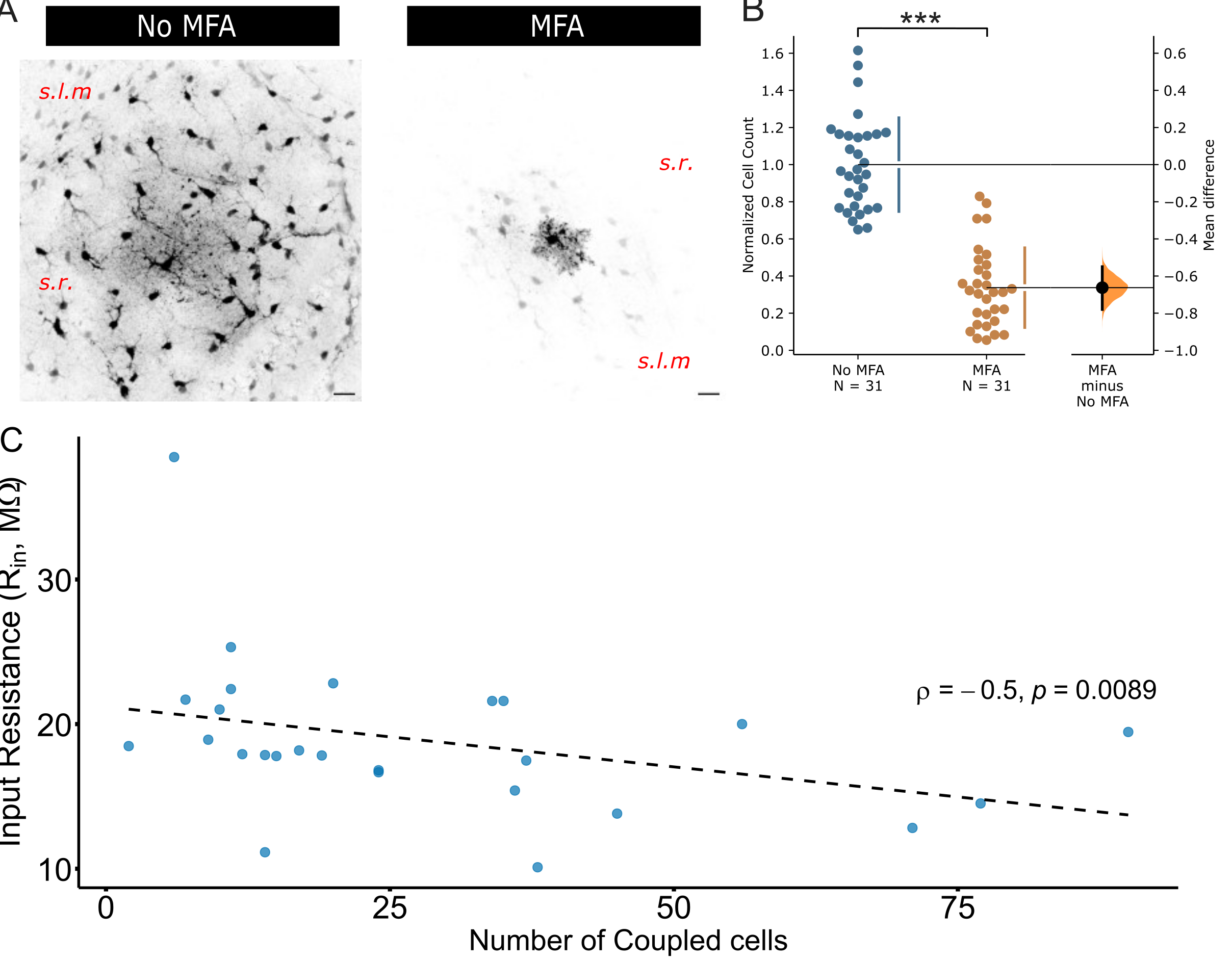

Supplement: Supplementary file 1 — Figure S1: Effect of MFA on dye and electrical coupling. (A) Left: example image of biocytin stained astrocytes in absence of MFA (No MFA) versus incubated in MFA (right). S.r., stratum radiatum; s.l.m., stratum lacunosum moleculare (scale bar = 20 μm). (B) Statistical summary of the coupled cell number in both groups. MFA reduces the number of stained astrocytes. Addition of MFA results in an average reduction of 71.4% [95% CI 60.0%, 83.7%], p < 0.00001 calculated for legacy purposes only. (C) Scatter plot illustrating the relationship between the physical size of the syncytium (number of biocytin‐coupled cells) and passive membrane input resistance (Rin) following gap junction blockade with MFA. A robust, statistically significant negative correlation is observed (Spearman's ρ = −0.502, p = 0.0089, n = 26). The inverse relationship between coupling size and input resistance (Rin) demonstrates that MFA‐induced reduction in dye transfer is accompanied by a concomitant decrease in electrical coupling. [file GLIA-74-0-s001.tiff]

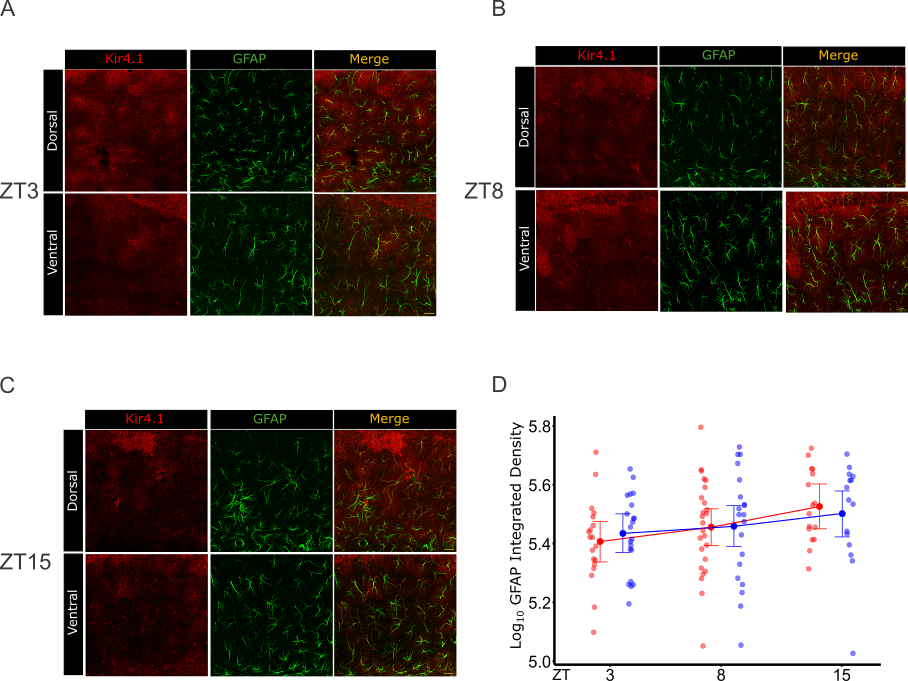

Supplement: Supplementary file 2 — Figure S2: Immunohistochemistry across times and regions. (A–C) Examples of confocal images of Kir4.1 (red) and astrocyte marker glial fibrillary acidic protein (GFAP, green), and the merging of the two images in the Stratum Radiatum of dorsal and ventral hippocampi across ZT3, ZT8, and ZT15, respectively. (D) Quantification of log10‐transformed GFAP integrated density across regions (dorsal hippocampus: red vs. ventral hippocampus: blue) and circadian times (ZT: 3, 8, 15). Only significant association was seen in the dorsal hippocampus where ZT15 shows 23.5% [95% CI 3.2, 39.2] more integrated density than at ZT3. [file GLIA-74-0-s002.tiff]
